# Combination of surface- and interference-enhanced Raman scattering by CuS nanocrystals on nanopatterned Au structures

**DOI:** 10.3762/bjnano.6.77

**Published:** 2015-03-17

**Authors:** Alexander G Milekhin, Nikolay A Yeryukov, Larisa L Sveshnikova, Tatyana A Duda, Ekaterina E Rodyakina, Victor A Gridchin, Evgeniya S Sheremet, Dietrich R T Zahn

**Affiliations:** 1 A. V. Rzhanov Institute of Semiconductor Physics, pr. Lavrentieva, 13, Novosibirsk 630090, Russia; 2 Novosibirsk State University, Pirogov str. 2, Novosibirsk 630090, Russia; 3 Novosibirsk State Technical University, pr. Karl Marx, 20, Novosibirsk, 630092, Russia; 4 Semiconductor Physics, Technische Universität Chemnitz, D-09107 Chemnitz, Germany

**Keywords:** copper sulfide (CuS) nanocrystals, interference-enhanced Raman spectroscopy, phonons, surface-enhanced Raman spectroscopy

## Abstract

We present the results of a Raman study of optical phonons in CuS nanocrystals (NCs) with a low areal density fabricated through the Langmuir–Blodgett technology on nanopatterned Au nanocluster arrays using a combination of surface- and interference-enhanced Raman scattering (SERS and IERS, respectively). Micro-Raman spectra of one monolayer of CuS NCs deposited on a bare Si substrate reveal only features corresponding to crystalline Si. However, a new relatively strong peak occurs in the Raman spectrum of CuS NCs on Au nanocluster arrays at 474 cm^−1^. This feature is related to the optical phonon mode in CuS NCs and manifests the SERS effect. For CuS NCs deposited on a SiO_2_ layer this phonon mode is also observed due to the IERS effect. Its intensity changes periodically with increasing SiO_2_ layer thickness for different laser excitation lines and is enhanced by a factor of about 30. CuS NCs formed on Au nanocluster arrays fabricated on IERS substrates combine the advantages of SERS and IERS and demonstrate stronger SERS enhancement allowing for the observation of Raman signals from CuS NCs with an ultra-low areal density.

## Introduction

Investigations of Raman scattering in nanostuctures such as nanocrystals (NCs) are limited by a low Raman cross-section because of the very low scattering volume of the nanostructures. Surface-enhanced Raman spectroscopy (SERS) taking advantage of plasmonics leads to a remarkable increase of the Raman sensitivity as shown for several semiconductor NC types [1–13]. The SERS effect by longitudinal optical (LO) phonons of CdS in Ag–CdS composite nanoparticles in solution and on a solid substrate was demonstrated [1–2]. Resonant SERS enhancement by LO phonons of CdSe was observed in core–shell CdSe/ZnS NCs deposited on commercially available nanostructured Au substrates [3]. Later, the SERS effect by LO phonons in CdSe in CdSe/ZnS NCs was realized on non-ordered nanostructured Ag surfaces [4]. Very recently, Lee et al. [5] reported the observation of SERS by surface optical (SO) and LO phonon modes in a CdSe core and the transverse optical (TO) phonon mode in a ZnS shell of core–shell CdSe/ZnS NCs attached to the surface of a Au nanowire. The spectrum of optical and interface phonons was obtained from the analysis of SERS spectra of pure CdSe NCs, core–shell CdSe/CdS, and CdSe/CdZnS NCs deposited on Ag SERS substrates [6]. A prominent enhancement of Raman scattering by LO phonons was observed in Au-ZnO NC nanocomposites [7] and ZnO NCs covered by Ag [8] excited near resonance with the interband electronic transitions in ZnO NCs.

Anomalously enhanced Raman scattering by LO phonons in epitaxial GaN and ZnO NC thin films covered with Ag was also explained by SERS [9]. A pronounced 10^4^-fold SERS enhancement by surface optical phonons was observed for ZnO NCs excited in resonance with localised surface plasmon in Ag nanoclusters deposited on ZnO NCs and out of the resonance [10–11]. SERS by LO phonons of CdTe was investigated in mixed Ag-CdTe NCs with a controllable Ag nanoparticle/CdTe NC mixture ratio [12]. The first report on the observation of the SERS effect by optical phonons in CuS NCs on ordered arrays of Au nanoclusters fabricated in a nanolithography process was published recently [13]. CuS NCs were synthesised by using the Langmuir–Blodgett (LB) technique, which allows the formation of arrays of CuS NCs with variable areal density. It was established that the variation of Au nanocluster size and shape in the nanostructured Au arrays governs the local surface plasmon resonance energy enabling resonance SERS in absorbates deposited on the arrays [13–17]. Moreover, CuS NCs are resistant against intense laser excitation even under resonant conditions. This is important for micro-Raman experiments with the NCs on nanostructured Au arrays under excitation in the green spectral region. These two issues make CuS NCs an attractive model system for SERS investigations of inorganic analytes. As it was demonstrated in [18], extremely thin absorbing coatings deposited on an antireflection layer (typically 100 nm SiO_2_ on Si) exhibit interference-enhanced Raman scattering (IERS) thus providing an alternative opportunity to enhance the Raman response by phonons in absorbates.

In this paper we present the results of both IERS and SERS and their combination by optical phonons in CuS NCs deposited on metal and semiconductor structures aiming at a maximal enhancement of the phonon response from CuS NCs.

## Results and Discussion

### Conventional Raman scattering

**Figure 1:**
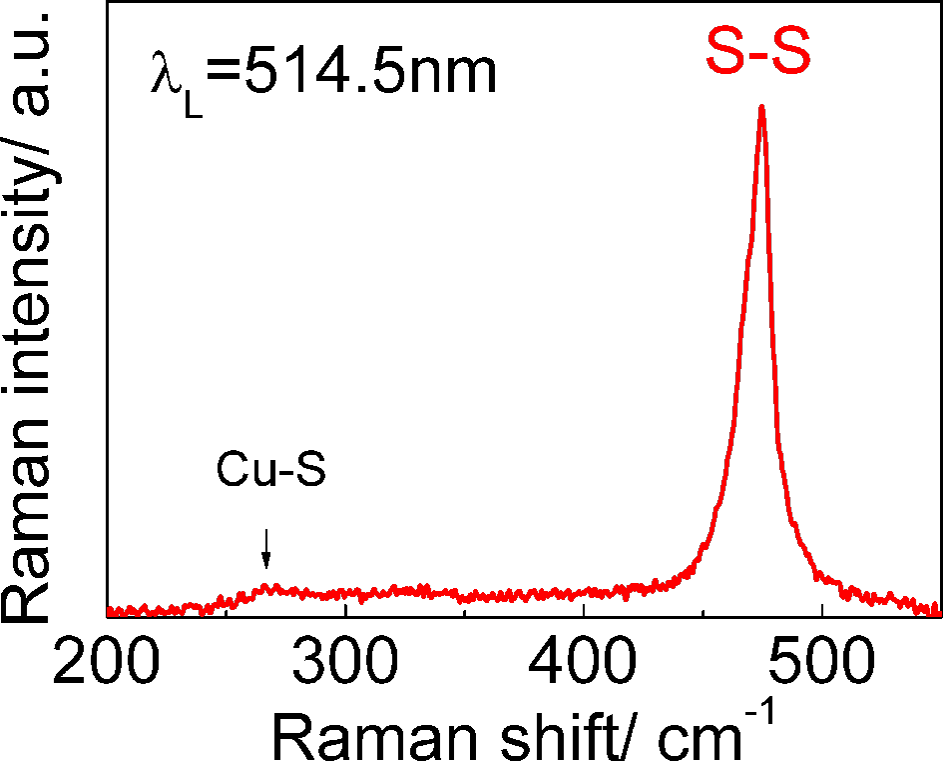
A typical Raman spectrum of the dense ensemble of CuS NCs (about 5–6 MLs) on a Au substrate excited with 514.5 nm.

A typical Raman spectrum of a dense array of CuS NCs deposited by LB technology on a Au substrate is shown in Figure 1. The average thickness of CuS NC film is about 32 nm, which corresponds to 5–6 monolayer (ML) coverage by CuS NCs. The spectrum reveals a pronounced peak at 474 cm^−1^, which is assigned to vibrational (stretching) modes from the covalent S–S bonds [19] and a much weaker peak at about 270 cm^−1^ attributed to the Cu–S bond vibration [19]. Therefore, the main attention was paid to the analysis of the Raman intensity of the most intense mode at 474 cm^−1^.

### Interference-enhanced Raman scattering

It was already established that thickness and refractive index of the SiO_2_ layer determine conditions for laser light interference, and thus for IERS by absorbates deposited on the sample surface [18,20]. Since these two parameters are crucial for the enhancement of the Raman scattering by the absorbing NC layer, we used spectroscopic ellipsometry to determine the precise value of the SiO_2_ layer thickness that gives the maximal IERS signal. The data on optical properties of CuS NC layer are also absent in literature, therefore, a wedge-shaped SiO_2_ layer with the thickness varying in the range from 0 to 570 nm was fabricated. This sample served as a substrate for the deposition of CuS NCs by using the LB technique and for further Raman experiments.

**Figure 2:**
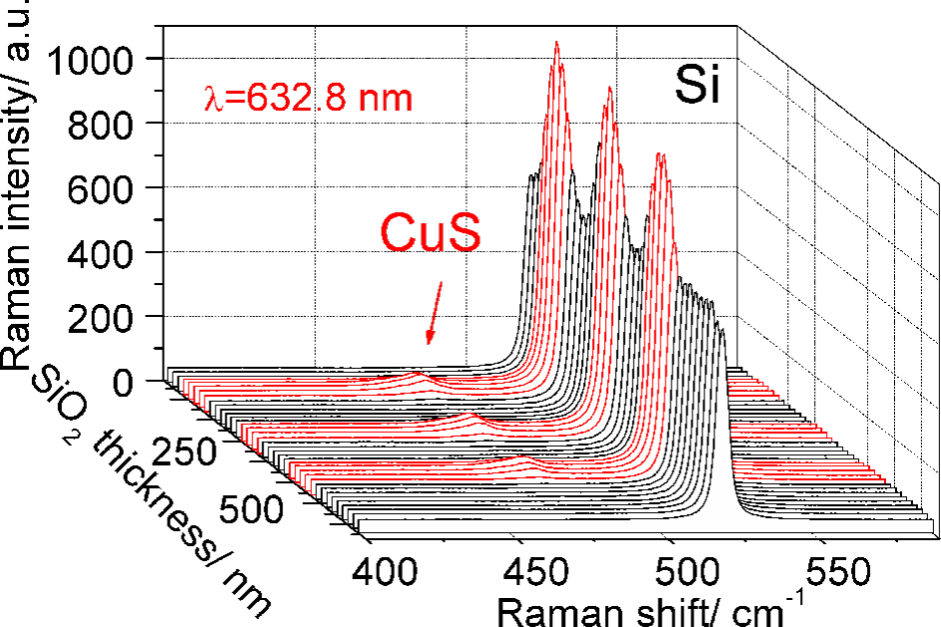
Raman spectra of CuS NCs (of about 1 ML coverage) fabricated on a Si substrate with a SiO_2_ layer of variable thickness. Raman spectra were taken using 514.5 nm excitation wavelength.

**Figure 3:**
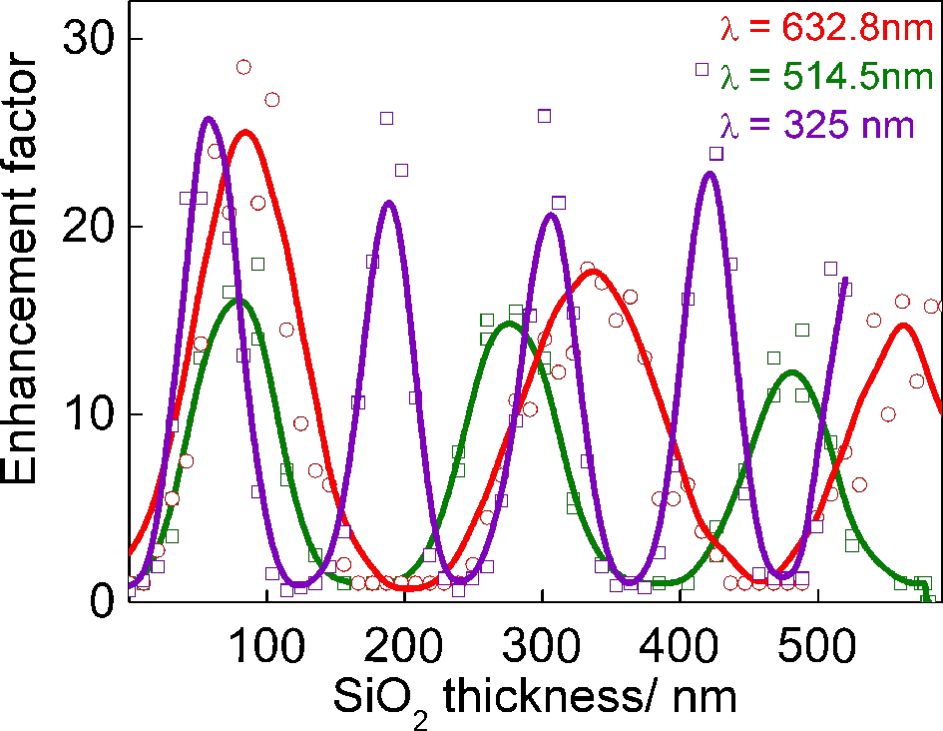
The dependence of the IERS enhancement factor of phonon modes in CuS NCs on the thickness of the SiO_2_ layer determined for laser excitation lines at 632.8, 514.5, and 325 nm.

The micro-Raman scattering study was performed in a backscattering geometry using a moving stage for precise positioning along the wedge-shaped sample in the direction of increasing thickness. Figure 2 shows the Raman spectra of CuS NCs recorded from areas with different SiO_2_ thickness. A significant periodic enhancement of the Raman scattering intensity by the phonon mode of CuS NCs indicates the presence of the IERS phenomenon. As the interference conditions inside the transparent oxide layer for the incident light wavelength changes with oxide thickness, a noticeable enhancement of the Raman scattering by the Si phonon (about 30%) also takes place. The periodic variation of the enhancement factor of the phonon mode in CuS NCs as a function of SiO_2_ layer thickness (Figure 3) was established for different laser excitation lines (632.8, 514.5, and 325 nm). Here, the intensity of the phonon mode of CuS NCs on a bare Si should be taken as a reference. However, it is worth mentioning that the intensity of the phonon mode is equal or below the noise level. Therefore, only an estimate of the IERS (as well as SERS) enhancement factor is possible. The maximal estimated IERS enhancement for the excitation wavelength of 632.8 nm reaches a value of at least 30 for a SiO_2_ layer thickness of 75 nm. As expected, the IERS enhancement maximum is observed for thicker SiO_2_ layers with increasing laser excitation wavelength. This SiO_2_ layer thickness (75 nm) was used for further combined IERS and SERS experiments.

### Surface-enhanced Raman scattering

**Figure 4:**
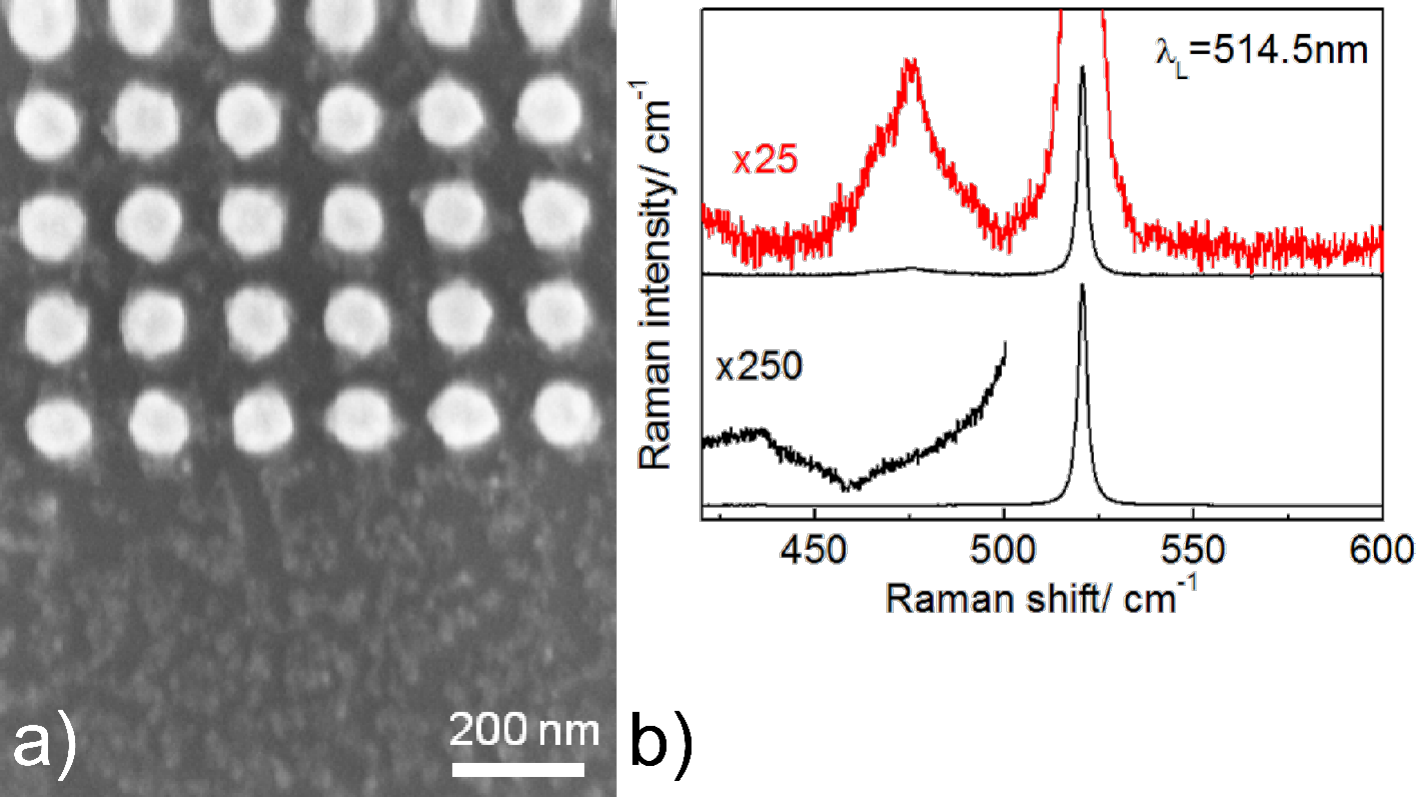
a) SEM image and b) micro-Raman spectra of CuS NCs deposited on Si (lower part) and Au nanocluster array (upper part).

A typical SEM image of the edge of a periodic Au nanocluster array on a Si substrate with deposited CuS NCs is presented in Figure 4a. One can see that the CuS NCs are homogeneously distributed on the Au nanocluster array and on the bare Si surface with an average thickness of about 1 ML. The Raman spectrum measured from the area where CuS NCs are formed on bare Si (lower part in Figure 4a) shows only one strong Raman line at 521 cm^−1^ related to the Si substrate (lower curve in Figure 4b). Weak Raman features located in the spectral range of 400–500 cm^−1^ are also typical for monocrystalline Si [21] while no evidence of the phonon modes from CuS NCs is detected. However, a strong Raman band centred near 474 cm^−1^ arises when the Raman spectra are acquired from the area where CuS NCs are deposited on the nanocluster array. The SERS enhancement factor can hardly be determined since no reference signal from CuS NCs is detected on bare Si. However, depending on the NC areal density the SERS enhancement factor was estimated as 30–50, which is comparable to that of IERS.

### Combination of SERS and IERS

**Figure 5:**
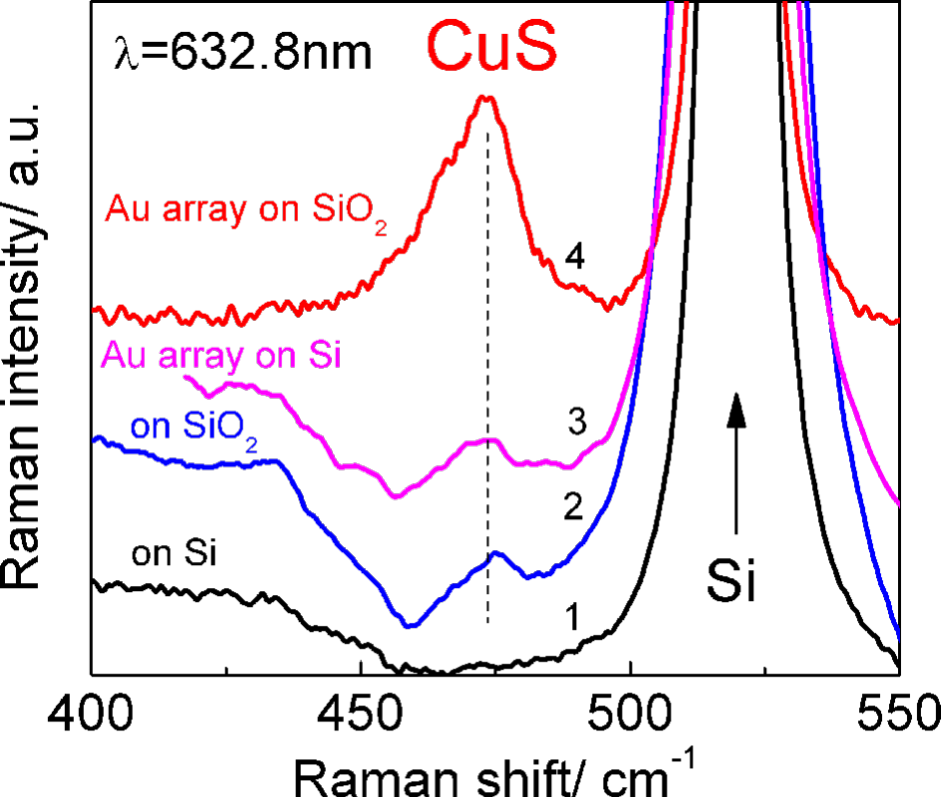
Raman spectra of CuS NCs deposited on bare Si, 75 nm SiO_2_ layer on Si, and on Au arrays fabricated on Si and SiO_2_ layer on Si measured with 632.8 nm.

In order to achieve even stronger enhancement of Raman scattering by phonons in CuS NCs by both IERS and SERS, CuS NCs were deposited on arrays of Au nanoclusters fabricated using nanolithography on a 75 nm thick SiO_2_ layer. Obviously, the LSPR energy of the Au nanocluster arrays fabricated on a SiO_2_ layer and on a Si substrate can be different due to the difference of dielectric functions of SiO_2_ and Si [22–23] that determine the LSP energy. Indeed, the CuS NCs on Au nanocluster arrays fabricated on a Si substrate excited with 514.5 nm demonstrate a prominent SERS signal from optical phonons of CuS NCs with the same effective coverage (of about 1 ML) (Figure 4), while no signal is detected when Au nanoclusters were fabricated on a SiO_2_ layer (not shown here). Most probably the shift of the LSPR energy in Au arrays fabricated on SiO_2_ layer is responsible for the absence of CuS phonon modes in Raman spectra excited with 514.5 nm. The situation changes when the Raman scattering of the same structures was investigated under excitation with a red laser line (632.8 nm). CuS NCs on Au nanocluster arrays fabricated on Si exhibit noticeable SERS signal with an intensity comparable with that of the IERS signal when CuS NCs are deposited on a bare 75 nm thick SO_2_ layer or an array of Au (Figure 5, curves 2 and 3, respectively). Note, that again no Raman signal is detected for NCs deposited on a bare Si substrate (Figure 5, curve 1). However, CuS NCs on Au nanocluster arrays fabricated on a SiO_2_ layer (Figure 5, curve 4) reveal further significant enhancement of Raman scattering (a factor of about 6). According to [24] the electromagnetic field has a maximum in the vicinity of an adsorbate/oxide interface due to constructive interference. The SERS intensity is proportional to the forth order of electromagnetic field and, therefore, can be significantly enhanced for the adsorbate (or CuS NCs) located in the local field. The constructive interference in oxide layers was successfully used for achieving the maximum ﬁeld enhancement for optical antennas [25], for realising the effect of co-enhanced IERS and SERS by organic molecules [26] and graphene [27], as well as for designing a chip for single molecular detection [28]. Thus, the use of the combination of SERS and IERS is preferable for the detection of a weak Raman response from a tiny amount of a material such as CuS NCs of an ultra-low areal density.

**Figure 6:**
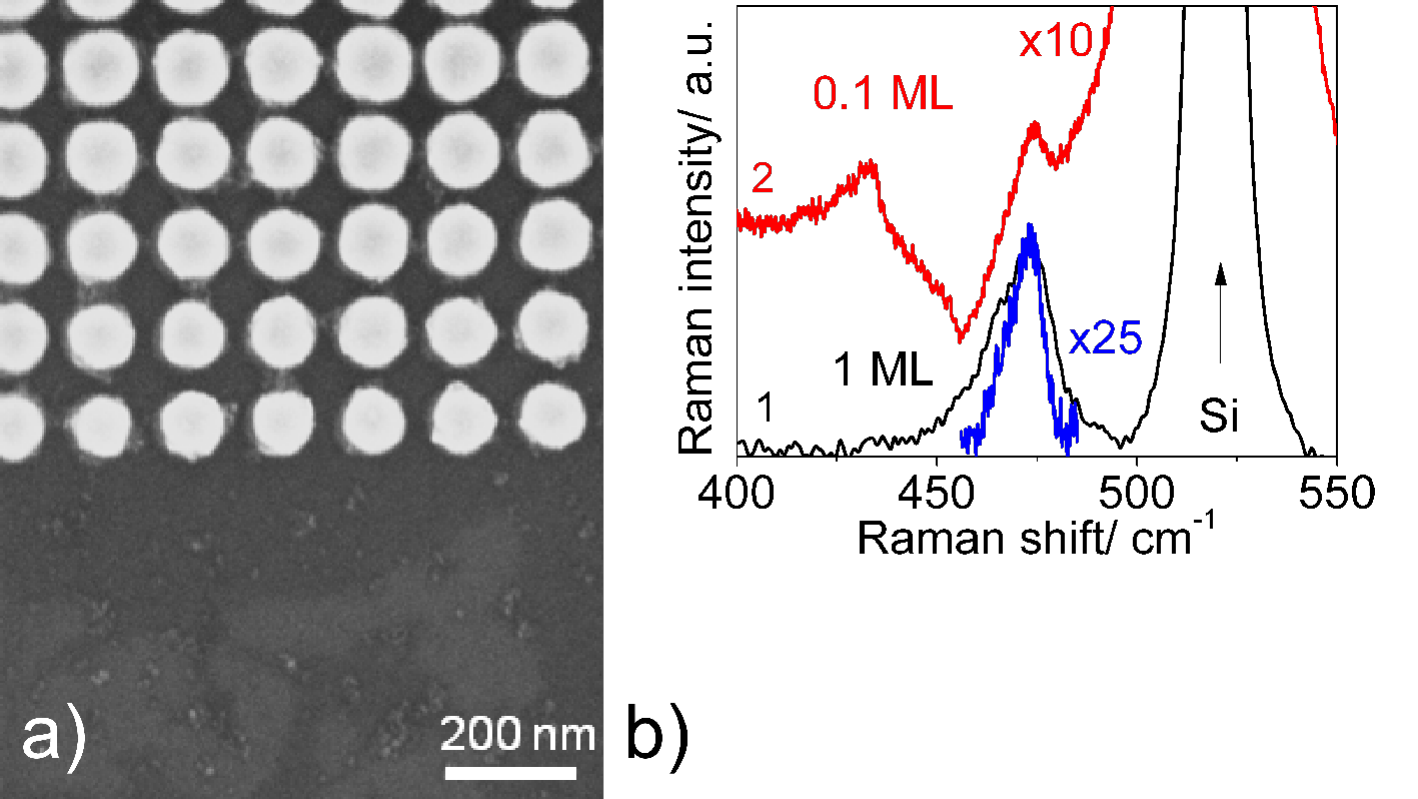
a) SEM image of CuS NCs with an ultra-low areal density deposited on Au arrays on SiO_2_ layer and b) SERS-IERS Raman spectra measured with 632.8 nm. SERS spectrum of CuS NCs with 1 ML coverage on Au arrays formed on SiO_2_ layer (curve 1) together with the Raman response of the CuS NCs of ultra-low areal density (denoted as 0.1 ML, curve 2). The 25 times enlarged fragment of curve 2 is shown for comparison.

Figure 6 shows a SEM image of homogeneously deposited CuS NCs with an areal density about ten times lower than that determined for the sample presented in Figure 4a. The SERS spectrum of the CuS NCs with ultra-low areal density shows a Raman phonon response at the same frequency position (about 474 cm^−1^) with an intensity about 25 times weaker than for 1 ML of CuS NCs. This low intensity is explained by the smaller number of CuS NCs taking part in the SERS process. It is important to emphasise that the full width at half-maximum (FWHM) decreases from 17 to 9 cm^−1^ with decreasing areal density. This can be explained by the decreasing interaction between individual CuS NCs in the ensembles with the ultra-low areal density and by the reduced number of agglomerates of CuS NCs.

## Conclusion

A periodical enhancement of Raman scattering by optical phonons from one monolayer of CuS nanocrystals fabricated by the Langmuir–Blodgett technology on a SiO_2_ layer deposited on a Si substrate was observed with the variation of the SiO_2_ layer thickness due to interference-enhanced Raman scattering. The pronounced enhancement of the Raman scattering from the nanocrystal ensembles deposited on arrays of Au nanoclusters evidences the surface enhanced Raman scattering effect. The combination of interference- and surface-enhanced Raman scattering led to a stronger enhancement of the phonon response by a factor of at least 180, and was successfully applied for probing the phonon spectrum of CuS nanocrystals with an ultra-low areal density.

## Experimental

The layer of thermally grown SiO_2_ with gradually varying thickness (from 0 to 570 nm) was prepared on a Si substrate in a wet chemical process by controlled dipping of a Si substrate covered by a homogeneous 600 nm thick SiO_2_ layer into HF solution in H_2_O (with bulk ratio 2:5) and served as IERS substrate. The thickness of the SiO_2_ layer was determined from spectroscopic ellipsometry measurements averaging over an area of about 1 mm.

Periodic Au nanocluster arrays with a size of 10 × 10 µm^2^ were fabricated as reported previously [13] on Si and 75 nm thick SiO_2_ layers by direct electron beam writing (Raith-150, Germany) of a 130 nm spin-coated resist ﬁlm (polymethyl methacrylate 950 K). A 40 nm Au ﬁlm with a 5 nm Ti underlayer for better adhesion was deposited by electron beam evaporation on the patterned positive resist. Finally, the resist was removed by a lift-off process in dimethylformamide resulting in a periodic array of Au nanoclusters. As a result, the fabricated SERS-active substrates consist of areas of Au nanoclusters with a period of 150 nm.

CuS NCs were fabricated on the IERS and SERS substrates using the Langmuir–Blodgett technology as reported in [29]. Briefly, at the first stage behenic acid dissolved in hexane was spread onto the water surface in a LB bath using a CuSO_4_ solution as a subphase. The copper behenate films formed were then transferred (Y-type) from the water surface onto a bare silicon substrate or SERS-active substrates. The typical thickness of copper behenate films ranged from 200 to 4 monolayers (MLs) corresponding to an average NC film thickness from 33 to 0.7 nm. At the second stage, the nucleation of CuS NCs in the organic matrix took place by sulfidizing the samples. At the last stage, 4 h of annealing at a temperature of 150 °C under argon atmosphere resulted in the removal of the organic matrix and the formation of free-standing CuS NCs on the substrates.

The morphology of the samples was determined using scanning electron microscopy (SEM). SEM images were obtained using a Raith-150 system at 10 kV acceleration voltage, 30 μm aperture and 6 mm working distance. It was found that the size of Au nanoclusters varied from array to array from 20 to 130 nm, while the height is fixed to 55 ± 5 nm. CuS NCs have a spherical shape and an average size in the range of 5–8 nm.

Raman experiments were carried out using Horiba T64000 and Labram HR800 spectrometers equipped with microscopes (the laser beam was focused to a spot with a size of 1 µm^2^) in a backscattering geometry at room temperature. He-Cd, Ar^+^, DPSS Cobolt^®^, and He–Ne lasers with wavelengths of 325, 514.5, 514.7, and 632.8 nm, respectively, and power below 2 mW were used for excitation.
